# Necrosis as a reaction to a wasp sting: a case report

**DOI:** 10.1590/S1678-9946202567073

**Published:** 2025-10-13

**Authors:** Abraão Aires Urquiza Carvalho, João Vitor Parente Mendes, Cristine Hirsch, Marcelo Dantas Tavares de Melo

**Affiliations:** 1 Universidade Federal da Paraíba, João Pessoa, Paraíba, Brazil; 2 Universidade Federal da Paraíba, Departamento de Ciências Biomédicas, João Pessoa, Paraíba, Brazil; 3 Universidade Federal da Paraíba, Departamento de Medicina Interna, João Pessoa, Paraíba, Brazil

**Keywords:** Wasp sting, Necrosis, Unusual reaction, Insect bite complication

## Abstract

A 62-year-old Brazilian woman with type 2 diabetes mellitus developed necrosis in her right arm following a single wasp sting. Severe reactions such as hers are typically associated with multiple stings and often manifest as anaphylactic shock rather than necrosis.

## INTRODUCTION

Wasps inject venom during stings to paralyze their prey or to protect their nest, comprising enzymes, amines, peptides, and other substances stored in their venom sacs. Key enzymes include phospholipases, which hydrolyze cell membrane phospholipids causing cell lysis, and hyaluronidase, degrading hyaluronic acid in the extracellular matrix. The venom’s amines, such as histamine, serotonin, and catecholamines, contribute to increased vascular permeability, pain, and tachycardia. Additionally, the venom contains diverse peptides that mediate various functions, including mast cell degranulation and stimulation of nociceptors^1^. Common reactions to wasp stings include local swelling, pain, and erythema. Some individuals with specific IgE antibodies against venom components may experience anaphylactic reactions shortly after being stung. A fourth, less common reaction includes skin rashes and serum sickness-like symptoms, potentially mediated by immune complexes or delayed hypersensitivity reactions^1^.

Occurrence of intense, widespread necrosis as a reaction to a wasp sting is exceptionally rare. This article reports the case of a 62-year-old woman, previously diagnosed with type 2 diabetes mellitus, who developed widespread necrosis in her right arm following a single wasp sting.

### Ethics

Informed consent was obtained from the participant involved in the study. The local ethics committee reviewed and approved the study under CAAE: 79014824.7.0000.5188.

## CASE REPORT

**Figure 1:**
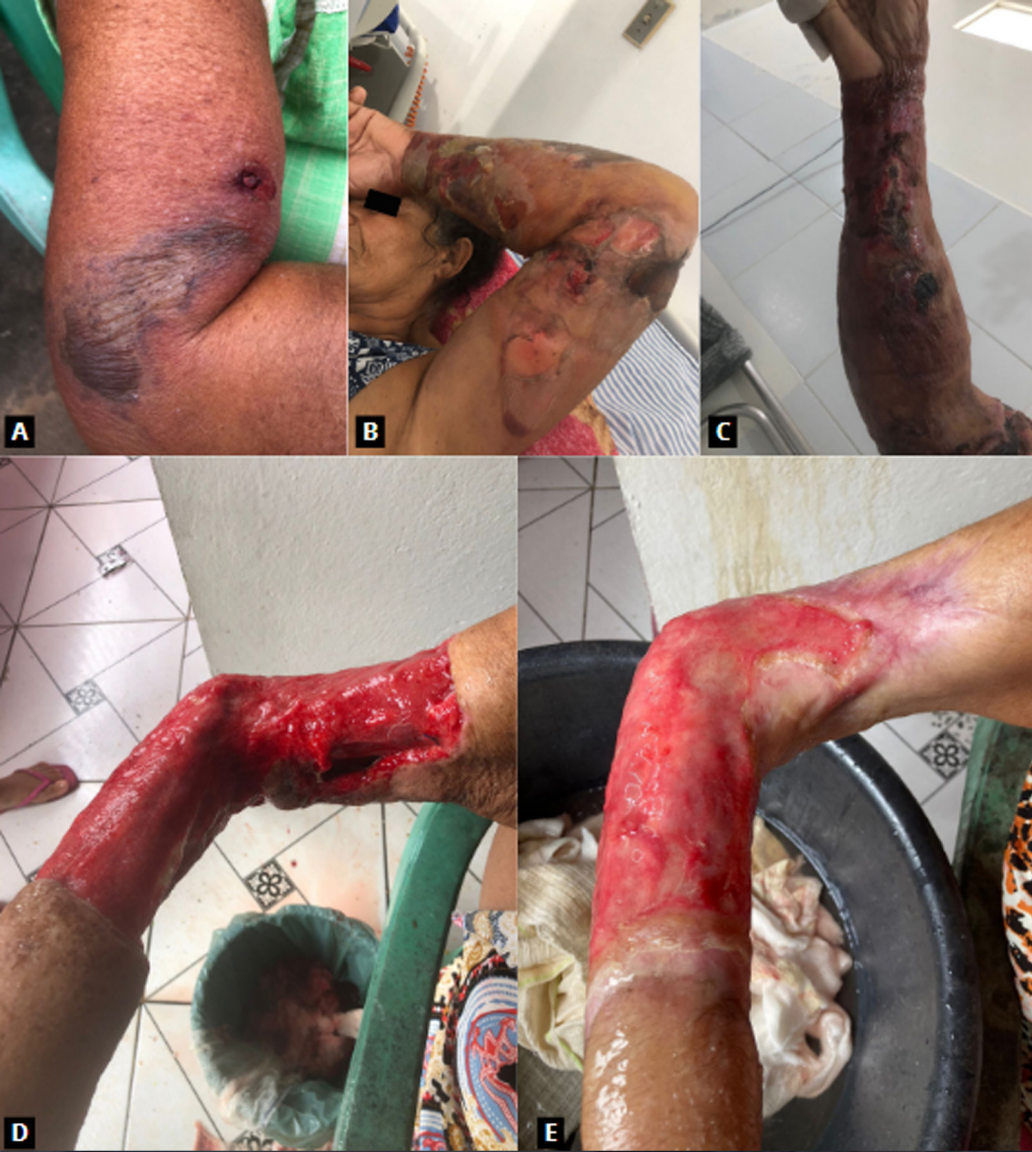
Different stages of the injury: (A) 5 days after sting; (B and C) 16 days after sting; (D) 20 days after debridement; (E) 3 months after debridement.

**Figure 2:**
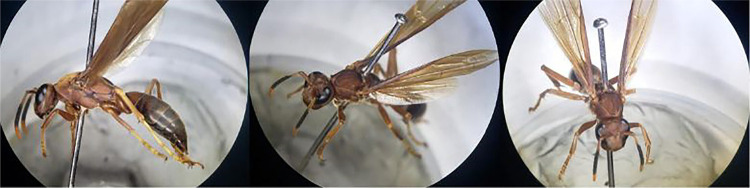
Specimen captured in Serraria, Paraiba State, at the site of the accident, 10 months after the event, identified as *Polistes canadensis*.

A 62-year-old Brazilian woman, diagnosed with Type II diabetes and with no previous history of atopy, residing in Serraria, Paraiba State, Brazil, sought emergency medical attention at a local hospital in March 2023 due to severe pain and edema in her right arm, accompanied by mild signs of localized necrosis (Figure 1A). The patient reported an incident occurring five days earlier where she was stung by a wasp, which she identified as “red wasp” or “red-headed hornet,” later identified as *Polistes canadensis* (Linnaeus, 1758) (Figure 2). Despite the lack of definitive diagnostic conclusions, she received an unspecified symptomatic treatment injection and was discharged. Her condition deteriorated the following day, prompting her to visit another hospital. There, she was treated with clindamycin (300 mg every 6 h), ciprofloxacin (500 mg every 12 h) for 10 days, and a single dose of ketoprofen (320 mg) administered for five days. Despite completing the treatment, her condition significantly worsened (Figures 1B and 1C). She was subsequently admitted to another hospital for further diagnostic evaluation and treatment. Remarkably, there was no evidence of pus formation nor fever. A hemogram was also performed, showing a hemoglobin of 12.9 g/dL, 167,000 platelets/mm^3^, and 20,700 leukocytes/mm^3^ with 17,388 segmented/mm^3^.

She remained hospitalized for five days, initially receiving intravenous clindamycin, followed by meropenem. After this period, she was transferred to another hospital for surgical debridement. During admission, her plasma glucose was 242 mg/dL, and she remained hospitalized for another 21 days. During this time, treatment with meropenem was continued at the same dosage for the first seven days, being replaced by cefepime (1 g every 8 h) for the remainder of her hospitalization.

**Figure 3:**
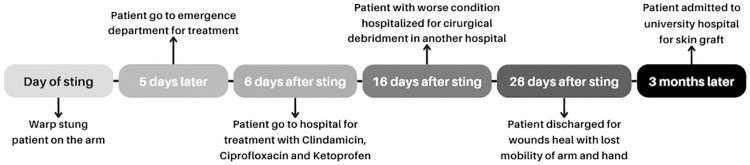
Timeline from the moment of the accident until the patient’s skin graft.

In total, the patient was hospitalized for 26 days, split between two different hospitals, treated with clindamycin, meropenem, and cefepime, and subjected to two surgical debridement until she was eventually considered clinically stable for discharge to allow the wound to heal in an outpatient basis (Figure 3). All this process resulted in significant loss of soft tissue in her right arm and loss of mobility in her arm and hand (Figure 1D).

Three months after her hospital stay, (Figure 1E), she was admitted to the university hospital for a skin graft procedure, where we first found out about her case.

## DISCUSSION

The Hymenoptera order, encompassing bees, bumblebees, wasps, and ants, represents one of the four largest insect orders globally. It boasts nearly 150,000 described species, with estimates projecting up to 2.5 million species worldwide. These insects exhibit diverse lifestyles, ranging from phytophagous to predatory and parasitic, across various ecological niches and environments^2^. In northeastern Brazil, Hymenoptera insects, particularly the wasp *Polistes canadensis*, have adapted well to urban environments^3,4^.

In the United States, venomous animal-related fatalities have been on the rise over the last six decades, with the Hymenoptera order being a significant contributor. From 2008 to 2015, an average of 86 annual deaths were related to venomous animals, 70% of which attributed to Hymenoptera stings^5^. Between 2009 and 2019, Brazil registered over 1.95 million incidents and nearly 3,000 deaths caused by venomous animals. Hymenoptera stings accounted for 7.4% of these incidents and 14.31% of the deaths, with a fatality rate of 0.29% and an annual average incidence rate of 6.89 per 100,000 inhabitants. Children aged 1-9 years experienced the highest incidence of accidents, whereas older adults had the highest fatality rate. Mild adult cases, unlikely to lead to systemic or anaphylactic shock, are often underreported^6^. Globally, the percentage of adults recalling at least one Hymenoptera sting varies by climate ranging from 56.6% to 94.5%, with insect sting mortality rates between 0.03 to 0.48 per million inhabitants per year^7^.

Cell death manifests with different morphological changes that have historically been used to classify it into three different forms: type I cell death or apoptosis, type 2 cell death or autophagy and type 3 cell death or necrosis. Although this morphological classification is still widely used, other forms of classification are being proposed, centered more around genetic, biochemical, pharmacological, and functional aspects involved in cell demise^8^. Apoptosis is a mode of programmed cell death characterized by extensive membrane blebbing, chromatin compaction and nuclear fragmentation, and play important roles in tissue homeostasis, morphogenesis, and timely removal of infected or injured cells. Necrosis, in turn, typically involves rapid cell swelling, producing a “ballooned” morphology that culminates in cellular lysis. This form of cell death is often associated with infections or sudden deviations from tissular homeostasis due to toxins, extreme temperatures, or trauma, but programmed forms of necrosis like necroptosis, ferroptosis and pyroptosis can also occur in specific circumstances^9^.

Necrosis can also be subdivided based on their microscopic and macroscopic appearances. Coagulative necrosis is often caused by ischemia of most organs (except the brain) and is characterized by the preservation of tissue architecture, with anucleate and eosinophilic cells. Liquefactive necrosis is associated with most infections (generating pus) and with ischemia of the central nervous system, and is defined by the loss of tissular structural integrity after cell digestion by hydrolytic enzymes, producing a liquified mass. Gangrenous necrosis is not a morphological classification, but instead a common clinical term to describe ischemic necrosis of the limbs. If the gangrene evolves with coagulative necrosis alone, it is called dry; if a superimposed bacterial infection produces liquefactive necrosis as well, the gangrene is considered wet. Apart from these three, necrosis can also be considered caseous (mostly associated with tuberculosis), fat (occurs in pancreatitis), or fibrinoid (a form of necrosis of the blood vessels caused by deposition of immune complexes) based on their macroscopic and microscopic aspects^10^.

Studies have reported cases of wasp stings causing tubular necrosis and myonecrosis^11^ or skin and soft tissue necrosis^12,13^, like here. Wasp venom could cause such events due to its innate myotoxic activity, disrupting muscle cell membrane through PLA1 and melittin. Mastoparan, a group of membrane-active amphipathic peptides, also induces myonecrosis, apoptosis, and cytokine activation by causing membrane destabilization and cell lysis^1^. Additionally, this necrosis could also be used as a possible clinical marker for worse prognosis. Yanagawa *et al*.^12^ highlighted that skin hemorrhage or necrosis was present in 13 out of 14 cases of rhabdomyolysis after wasp stings analyzed by the authors, suggesting that such occurrences may be associated with worse outcomes.

Wasp stings could also cause compartment syndrome, either due to local edema mediated by the venom itself^14^or secondary bacterial infection^15^. Wasps also seem more likely to represent a microbial hazard than honeybees. Gkitsak *et al*.^16^ used stinging stimulation experiments for microbial recovery and identification from hymenopterans, and found that wasps and hornets seem to be in contact with important hygienic indicators (i.e., enterococci, *Proteus mirabilis*, and coliforms) and pathogens like *Enterobacter spp*. and *Klebsiella spp*., known for multidrug resistance.

Our patient’s glycemic control could have also played a role in her unfavorable evolution. A retrospective cohort study compared the infection rates between diabetic patients and non-diabetic control, and found that diabetic patients had higher rates for all infections, with the highest IRR’s being in bone, joints, cellulitis, and sepsis. Overall IRR for infection-related hospitalization was 1.88 for type 2 diabetes. This higher incidence ratio has been linked to a variety of reasons, such as impaired immune response due to hyperglycemia, diabetic neuropathy, altered lipid metabolism, and others^17^. Additionally, diabetic patients also have poor wound healing associated with the damage to the microcirculation, excessive inflammation, and reduced angiogenesis characteristic of diabetes^18^.

## CONCLUSION

We reported a case of a diabetic patient that evolved with severe necrosis after a single wasp sting, causing an important loss of mobility in the affected limb. Our report has some important limitations. We could not access the patient’s glycemic control status, including feasting plasma glucose and HbA1c levels, prior to the event, allergen-specific IgE testing nor any form of histopathological examination of the necrotic wound, bacterial culture or antibiogram. As such, we cannot report with certainty the type of necrosis present or which microorganisms were involved in the process. We also could not get in contact with the medical team that treated our patient during her hospital stay, and the information reported here was extracted from her medical records, both during her hospital stay and in primary care, and also from her own report. Thus, we could not access the rationale behind her antimicrobial treatment. Regardless of the causes, hymenopteran accidents have been on the rise lately, and physicians should be aware that wasp stings can, although rarely, lead to necrosis. Timely diagnosis and intervention are crucial to decrease morbidity and mortality since this form of lesion may rapidly progress.

## Data Availability

The complete anonymized dataset supporting the findings of this study is included within the article itself.
